# Cause-and-effect relationships in a nonlinear model of Bitcoin’s energy use and price volatility effect

**DOI:** 10.1371/journal.pone.0334537

**Published:** 2025-10-17

**Authors:** Georgia Zournatzidou

**Affiliations:** Departmenf of Business Administration, University of Western Macedonia, Grevena, Greece; University of Patras: Panepistemio Patron, GREECE

## Abstract

The environmental impact of Bitcoin (BTC) has been a source of concern due to its substantial energy consumption, which is a result of its proof-of-work mining algorithm and transaction processes. The global usage levels of Bitcoin are comparable to those of some affluent nations. This study examines the nonlinear causal relationship between the energy consumption of Bitcoin and its price volatility. In order to evaluate causality, we implement a nonlinear Granger causality test that is bolstered by artificial neural networks (ANNs). These networks are capable of recognizing intricate, nonlinear relationships that conventional linear models may be unable to identify. Our results indicate a substantial causal relationship between the price volatility of Bitcoin and fluctuations in its energy consumption, indicating that energy usage patterns can be used as indicators of market behavior. These findings have significant implications for regulators and investors, under-scoring the necessity of monitoring energy consumption trends to gain a more comprehensive understanding of the Bitcoin market dynamics and to inform policy decisions.

## 1. Introduction

Since its introduction by Satoshi Nakamoto in 2008 [1], Bitcoin has evolved from a niche digital asset to a critical component of the global financial system. Its rise is largely attributed to its decentralized structure, underpinned by blockchain technology and the absence of central regulatory authority. Despite its growing prominence, Bitcoin’s accelerated adoption has sparked significant concerns, particularly related to its extreme price volatility and substantial energy consumption. Hairudin et al. [2] conclude that public acceptance of cryptocurrencies remains limited, with many individuals hesitant to adopt them as alternatives to traditional fiat currencies.

Bitcoin’s price volatility is a key feature of its market behavior, characterized by abrupt and substantial fluctuations over short periods [3–6]. This volatility is influenced by a range of factors including speculative trading, regulatory developments, macroeconomic conditions, and market sentiment [7]. This inherent unpredictability in the Bitcoin market presents challenges for investors, regulators, and policymakers alike. Previous studies have demonstrated that Bitcoin’s price is highly sensitive to geopolitical events and speculative activities, with the volatility exacerbated by the cryptocurrency market’s nascent stage and relatively low liquidity compared to traditional financial markets [8]. Researchers such as Urquhart [9] and Blau [10] have examined the inefficiency of Bitcoin markets and the role of speculative trading, respectively, as primary drivers of this volatility. Furthermore, studies by Tan et al. [11] and Omura et al. [12] have employed advanced econometric models to forecast Bitcoin’s volatility, highlighting the complex interaction of multiple market forces [6,10,13]. Parallel to concerns over volatility, Bitcoin’s energy consumption, driven by the Proof-of-Work (PoW) consensus mechanism, has become a key point of environmental debates [14]. PoW ensures transaction validity and network security but demands considerable computational power [15]. Estimates indicate that Bitcoin’s energy consumption is comparable to that of some smaller nations [16,17], raising sustainability concerns and prompting calls for alternative consensus mechanisms or improvements in mining efficiency [18–20].

De Vries [16] and Gallersdörfer et al. [21] emphasize Bitcoin mining’s significant carbon footprint, predominantly due to its reliance on fossil fuels. However, Mora et al. [22] and Sedlmeir et al. [23] suggest a gradual shift towards renewable energy sources, driven by economic incentives in regions rich in solar, wind, and hydropower resources. Geographical differences in mining practices also contribute to this dynamic; for instance, China historically relied on coal until regulatory restrictions shifted operations to more sustainable regions like North America and Northern Europe. Policymakers are increasingly intervening to promote sustainable mining practices through taxes, subsidies, and regulatory frameworks, as explored by Stoll et al. [18]. The interraction between energy markets and financial assets has been explored in various contexts, such as the impact of crude oil price fluctuations on firm performance [24]. Their study highlights how energy price volatility can influence market dynamics even in sectors indirectly related to energy consumption. This is particularly relevant for Bitcoin, where energy consumption is not just an operational cost but a fundamental component of the network’s functioning, suggesting that fluctuations in energy consumption may directly contribute to Bitcoin’s market volatility.

While extensive research has been conducted on Bitcoin’s price volatility [25] and its energy consumption, fewer studies have explored the interaction between these two dimensions. Early investigations have established correlations between Bitcoin’s price and mining-related variables, such as long-term cointegration with mining costs [26]. Krause and Tolaymat [27] quantified Bitcoin’s environmental impact, attributing high energy demands to the competitive nature of mining, which incentivizes the use of powerful hardware to maximize rewards.

Recent studies [28] have begun to explore the link between energy consumption and market behavior. For example, Corbet et al. [7] identified a positive correlation between electricity prices and Bitcoin returns, while Karmakar et al. [29] examined how Bitcoin mining activities influence volatility dynamics in U.S. electricity markets. Huynh et al. [30] examined the relationship between Bitcoin’s energy consumption and its market dynamics. Utilizing variance decompositions alongside realized semi-variances on daily data, they identify a connection between Bitcoin’s energy usage, returns, and trading volumes. Furthermore, their results indicate that over the long term, trading volumes have a more substantial impact on energy consumption than returns. The study also highlights the predictive power of energy consumption concerning Bitcoin’s returns and volume, emphasizing the need for sustainable innovations within the cryptocurrency ecosystem to mitigate environmental impacts. Moreover, Omura et al. [12] demonstrated how external factors like natural gas volatility can spill over into Bitcoin price volatility using the HAR-RV model.

Despite these advancements, a significant gap remains in understanding the *non-linear* causal relationships between Bitcoin’s energy consumption and its price volatility. Sapra et al. [31] highlighted the complexity of these interactions, suggesting that Bitcoin prices can influence energy use, but the reciprocal effect of energy consumption on volatility is less understood.

Addressing this gap, the present study investigates the non-linear causal relationship between Bitcoin’s energy consumption and price volatility. Unlike previous research that primarily employed linear models or singular volatility measures, we apply a multi-faceted approach using a variety of volatility estimators, including the Garman-Klass, Parkinson, and Rogers-Satchell measures. This allows for a comprehensive assessment of Bitcoin’s volatility characteristics.

Furthermore, we utilize nonlinear Granger causality tests, supported by artificial neural networks (ANNs), to detect complex, non-linear interactions that traditional econometric models may overlook. This methodology builds on the foundational work of Maiti [32], which suggested potential non-linear linkages between Bitcoin prices and mining costs, and extends the analysis to capture the broader interdependencies between energy consumption and market dynamics [32].

By exploring these non-linear relationships, our research contributes to the broader discourse on the sustainability and financial stability of Bitcoin. The findings have implications for investors, regulators, and policymakers, offering novel insights into how energy consumption patterns can inform market behavior and influence regulatory strategies.

The remainder of this paper is organized as follows: Section 2 details the methodology, including the volatility measures and nonlinear causality tests employed. Section 3 describes the data sources and preprocessing techniques. Section 4 presents the empirical results, followed by a discussion of their economic implications in Section 5. Finally, Section 6 concludes with key insights and suggestions for future research.

## 2. Methods

This section offers a comprehensive account of the mathematics used to derive the volatility estimate functions for the open, high, low, and closing values of cryptocurrencies, with a particular emphasis on Bitcoin. In this work, we use three distinct volatility estimators—Garman-Klass (vGK), Parkinson (vParkinson), and Rogers-Satchell (vRS)—to analyze different facets of Bitcoin price volatility. Each estimator is specifically crafted to assess volatility using distinct methodologies, utilizing varied pricing data inputs [33,34]. Specifically, vGK exploits the full OHLC set under (near) zero-drift/continuous-trading assumptions, delivering an efficient range-based estimator when opening jumps are limited—conditions that broadly fit 24/7 crypto trading. vParkinson relies solely on the daily high–low range and is highly efficient under diffusion without drift, but it can be biased by jumps and microstructure noise. vRS, by contrast, accommodates non-zero drift and directional moves, making it more reliable in trending markets. We compute each estimator from daily OHLC data and compare their signals; agreement strengthens inference, while divergences help diagnose whether volatility is range-driven (vParkinson), drift-sensitive (vRS), or balanced across inputs (vGK). Employing several estimators enables us to have a more thorough understanding of Bitcoin’s volatility characteristics. We further use the nonlinear causality approach to identify intricate nonlinear causal relationships between Bitcoin-based energy usage and Bitcoin volatility.

### 2.1. Volatility estimators

The first volatility estimator utilized is the Parkinson Volatility Estimator (Parkinson, 1980).


$$\hat{\sigma_{p}^{\text{2}}}=\frac{{\left(p_{max}-p_{min}\right)}^{\text{2}}}{\text{4}ln\text{2}}$$ (1)

Where, $p_{max}=\text{ln}(High)-\text{ln}(Open),$ and $p_{min}=ln(Low)-ln(Open).$ High, Open and Low refer to the High, Open and Low Bitcoin price data. This function is employed to quantify the volatility estimators of open prices for the objectives of this study. This estimator was selected exclusively for open prices due to its unique ability to capture the market’s precipitous response to overnight news and events. The Parkinson estimator is renowned for its ability to generate a more accurate volatility estimate than models that exclusively rely on closing prices, as it is particularly sensitive to extreme price movements (i.e., the highest and lowest points within a time frame).

The Garman-Klass Estimator [35] was employed to define an additional volatility estimator in response to the elevated Bitcoin prices. The Garman-Klass estimator is a sophisticated volatility measure that calculates volatility by utilizing the opening, closing, high, and low prices of an asset over a specific period. It is widely regarded as more efficient than fundamental measures that solely rely on closing prices because it incorporates a broader range of price data, including the intraday high and low, which often more accurately reflect market fluctuations. Define the Garman-Klass Estimator as follows:


$$\hat{\sigma_{GK}^{\text{2}}}=\text{0}.\text{5}{\left(p_{max}-p_{min}\right)}^{\text{2}}-(\text{2}ln\text{2}-\text{1})p_{close}^{\text{2}}$$ (2)

Where $p_{close}=ln(Close)-ln(Open)$. Close refers to the final Bitcoin values. The justification for using this estimator for elevated cryptocurrency prices is its design for assets characterized by high volatility, such as cryptocurrencies. This is due to its inclusion of the whole spectrum of price fluctuations (high, low, close) for a particular trading day.

Ultimately, the Rogers-Satchell (RS) estimator [36] is used for closing prices, calculated as follows:


$$\hat{\sigma_{RS}^{\text{2}}}=p_{max}\left(p_{max}-p_{close}\right)+p_{min}\left(p_{min}-p_{close}\right)$$ (3)

This volatility estimator was chosen for the purpose of closing Bitcoin prices due to its ability to accurately capture all intraday price fluctuations associated with both the opening and closing prices. Furthermore, the high, low, open, and close prices are incorporated into the formula, which enables it to accurately represent the volatility associated with the closing price [37]. When a distinct price trend is present, the Rogers-Satchell estimator is more effective than other estimators in capturing volatility. The Rog-ers-Satchell estimator may be able to capture the trend-driven volatility that frequently arises in Bitcoin, whether it be upward (bullish) or downward (bearish). The incorporation of this estimator allows us to account for the directional price movements of Bitcoin while still considering the intraday highs and lows [37].

### 2.2. Nonlinear Granger causality test

Granger causality has been validated as a dependable approach to identifying causal relationships. The application of Granger causality to various frequency bands is effective in determining the intensity and direction of causality, which can fluctuate across frequencies. The spectral-density approach was initially introduced by Granger [38,39] to provide a more detailed and concise illustration of causality than a singular Granger causality measure that was applied uniformly across all periodicities. Consequently, the traditional one-shot test is less efficient than the measurement of bivariate Granger causality over the spectrum. In this context, two critical issues arise: the first is the extent to which causality varies with frequency, and the second is the question of whether the significance and direction of standard Granger causality tests in the time domain vary when applied across various frequency bands.

Linear causality tests, including the Granger causality test, may have limited power to identify specific categories of non-linear causal relationships [40,41]. In simplified terms, these tests may overlook non-linear patterns that can predict future values. When applied to residuals from Vector Autoregression (VAR) models, two critical issues arise regarding the statistical properties of these tests. Baek and Brock [40] specifically contend that their modified test preserves the same asymptotic distribution regardless of whether the residuals are genuinely independent and identically distributed (iid) errors or consistently estimated residuals from a VAR model [40]. The testing procedure is simplified by this characteristic, which is referred to as nuisance-parameter-free (NPF). We anticipate that the modified test utilized in this investigation will exhibit comparable NPF characteristics. Secondly, Hiemstra and Jones [41] provide Monte Carlo evidence that suggests the modified test is robust to nuisance parameters, despite the NPF advantage [41]. Furthermore, they observe a correlation between the asymptotic and finite-sample properties of the test when it is applied to residuals estimated from a VAR model.

In this paper, we suggest a non-linear test that is based on Artificial Neural Networks (ANNs) to address the constraints of linear causality tests, as outlined in Hmamouche (2020). This method is designed to analyze non-linear relationships between time series data. Specifically, we present a non-linear extension of the Granger causality test that employs feed-forward neural networks.

ANNs are particularly well-suited for modeling nonlinear relationships due to their ability to approximate complex, non-linear functions without prespecified parametric forms. Their multi-layered architecture allows for the detection of intricate patterns within data, which traditional linear models may fail to capture. This capability is critical in financial time series analysis, where market behaviors often exhibit non-linear dependencies and dynamic interactions like the Bitcoin market. Within an extended Granger causality framework, we utilize a Vector Autoregressive Neural Network (VARNN) model to capitalize on the capabilities of ANNs. Subsequently, we offer a succinct overview of the VARNN model.

#### 2.2.1. Granger causality selection on encoding.

This study utilizes a Vector Autoregressive Neural Network (VARNN) model for predicting future values of a target variable (X). The model assumes a p-dimensional stationary time series observed at T time points and leverages a training dataset containing x and k predictor variables $\left\{x_{\text{1}},\ldots ,x_{k}\right\}.\;$The VARNN is a multi-layer perceptron (MLP) neural network, a common type of feedforward ANN. Actually, The Multi-Layer Perceptron (MLP) builds upon the foundational perceptron model introduced by Rosenblatt [42] and further developed by Widrow and Hoff [43]. An overview of the MLP is provided as follows: An MLP consists of a minimum of three layers of nodes, specifically (i) an input layer, (ii) a hidden layer, and (iii) an output layer. Except for the input nodes, each node functions as a neuron utilizing a nonlinear activation function. The MLP employs a supervised learning technique known as backpropagation for training purposes. For a more comprehensive discussion on the statistical modeling of artificial neural networks using MLP, we refer to the work of Aitkin and Foxall [44]. It incorporates lagged values of both the target variable and the predictors to predict future x values.

Our choice of VARNN allows us to tailor the prediction of each target variable to a specific set of predictors. Notably, different target variables may require different predictor sets. The model employs two hidden layers with sizes of two (univariate) and four (bivariate) neurons, respectively (as shown in equations 5 and 6). The VARNN initially reformats the data for supervised learning based on the chosen lag parameter. The model utilizes the stochastic gradient descent (SGD) algorithm with a learning rate of 0.1 to update the network weights during training. Following Hmamouche (2020), the model employs the rectified linear unit (ReLU) activation function in the hidden layers and the sigmoid function in the output layer.

In our empirical analysis, we employ the SGD algorithm. We next define the global function of the VARNN (p) as follows:


$$x_{t}=\psi_{nn}\left(x_{t-\text{1}},\ldots ,x_{t-p},\ldots ,x_{k(t-\text{1})},\ldots ,x_{k(t-k)}\right)+u_{t}$$ (4)

where $\psi_{nm}$ and $u_{t}$ represent the network function and the error terms, respectively.

Similar to Granger causality, we focus on the case of two variables, $x_{t}$ and $y_{t}$. To assess the causal influence of $x_{t}$ on $y_{t},$ we employ two prediction models. The first model predicts the target variable solely based on its own lagged values. The second model incorporates both lagged values of the target variable and lagged values of the predictor for prediction.


$$y_{t}=\psi_{\text{1},nn}\left(y_{t-\text{1}},\ldots ,y_{t-p},\ldots ,y_{k(t-\text{1})},\ldots ,y_{k(t-k)}\right)+u_{\text{1},t}$$ (5)

$$y_{t}=\psi_{\text{2},nn}\left(y_{t-\text{1}},\ldots ,y_{t-p},x_{t-\text{1}},\ldots ,x_{t-p}\right)+u_{\text{2},t}$$ (6)

where $\psi_{\text{1},nn}$ and $\psi_{\text{2},nn}$ refer to the network functions of the two models considered, respectively.

Causality testing involves F-statistics to assess whether lagged values of a predictor variable $x_{t}$ provide statistically significant information about a target variable $y_{t}$, even when lagged values of $y_{t}$ are already considered. In simpler terms, the F-test helps us determine if $x_{t}$ has a causal effect on $y_{t}$, beyond the influence of $y_{t}$’s own past values. The null hypothesis tested is that lagged $x_{t}$ do not Granger-cause series $y_{t}$.

## 3. Data

The sample period spans from July 5, 2019, to July 4, 2024, and the data pertains to the daily price of Bitcoin (Open, High, Low, and Close), with a daily sampling frequency. For the purposes of our analysis, we also employ daily data for the Bitcoin energy consumption, with the Cambridge Bitcoin-based Electricity Consumption (CCAF) serving as a proxy. Specifically, the Cambridge Bitcoin Electricity Consumption Index is a tool that has been developed to quantify the daily electricity consumption of the Bitcoin network. Logarithmic differentiation has been implemented for the objectives of our investigation.

The fundamental statistics of the Bitcoin-Based Electricity Consumption Index and each volatility estimator for Bitcoin are presented in Table 1. There are no series that are not being considered on a daily basis. As demonstrated by this table, the volatility measures (Garman-Klass, Parkinson, and Rogers-Satchell) exhibit comparable means of approximately 0.38, suggesting a moderate level of volatility. However, the volatility data may not be normally distributed, as evidenced by the presence of high skewness and kurtosis values, as well as significant Jarque-Bera statistics. The Bitcoin Electricity Consumption Index exhibits a mean of 36.51 and a relatively low standard deviation. However, it also exhibits negative skew-ness, which suggests a lengthier tail toward lower consumption values. Overall, the Jarque-Bera test rejected the null hypothesis of normality in all series under consideration, with the values of the test indicating a 1% significance level.

**Table 1:** Descriptive statistics for each volatility estimator (Garman-Klass, Parkinson and Rogers-Satchell), and Bitcoin-based energy consumption index.

	Garman-Klass volatility	Parkinson volatility	Rogers-Satchell volatility	Bitcoin-based electricityconsumption index consumption
	${vGK}_{t}$	${vParkinson}_{t}$	${vRS}_{t}$	${CBECI}_{t}$
Mean	0.3824	0.3819	0.3755	36.51
Median	0.3484	0.3434	0.3392	36.63
Maximum	1.7211	1.8894	1.7894	38.70
Minimum	0.0435	0.0404	0.0000	12.16
Std. Dev.	0.2037	0.2118	0.2084	1.2708
Skewness	1.4851	1.4076	1.6008	−6.2056
Kurtosis	7.0798	6.6422	7.6643	92.4211
J-B	1939.7^***^	1641.1^***^	2437.7^***^	620432
J-B Prob.	[0.0000]	[0.0000]	[0.0000]	[0.0000]
Obs	1827	1827	1827	1827

Notes: This table reports descriptive statistics for each volatility estimator (in logarithmic transformation), and the logarithm of the Cambridge Bitcoin-based Electricity Consumption Index daily series. The following statistics are given: Mean, median, maximum, minimum, standard deviation (Std. Dev), skewness, kurtosis, Jarque Berra normality test (J-B). The null hypothesis that the series is normally distributed is also tested by the Jarque-Bera test. The p-values of the test are given below in brackets. *** indicates a rejection of the null hypothesis of normality at a 1% significant level Fig 1.

**Fig 1:**
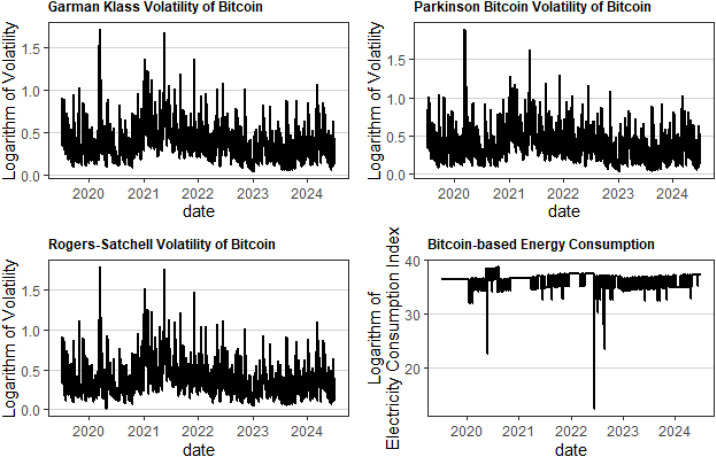
Logarithm of each volatility estimator calculated based on daily Bitcoin prices, as well as the Cambridge Bitcoin-based Electricity Consumption Index (logarithmic transformation).

## 4. Results

The findings in Table 2 indicate a potential causal relationship between the Garman-Klass Volatility of Bitcoin and the consumption of electricity based on Bitcoin. At latencies 1, 2, 5, 7, 14, 20, and 25, the null hypothesis (no causality) is rejected at the 1% level, as evidenced by statistically significant F-values (p-values in brackets) at various lag parameters. However, the significance level decreases for delays exceeding 20 days, indicating a diminished causal impact of electricity consumption on Bitcoin volatility. The volatility of Bitcoin (as measured by the Parkinson estimator) may be influenced by Bitcoin-based Electricity Consumption Index Consumption, as indicated by the results in Table 3. This is corroborated by statistically significant F-values for all delays, which range from one day to one month. The null hypothesis of no causality is rejected at the 1% level for these delays, which also suggests a robust relationship. Additionally, it is important to observe that the significance of the relationship between electricity consumption and volatility decreases as the latency increases beyond two weeks (14 days), which implies that the causal effect of electricity consumption on volatility is diminishing over time. In Table 2, we observe strong and pervasive evidence that past BEC helps explain subsequent volatility measured by vGK over short to medium horizons. For instance, at p = 1 the Granger causality index (GCI) is 3.38E-01 with an F-statistic of 72.67 (p < 10 ⁻ ¹²⁴), and at p = 2 the GCI rises to 5.35E-01 with F = 79.54 (p ≈ 2.0 × 10 ⁻ ¹⁹⁵). Predictive content remains sizable at p = 5 (GCI = 6.63E-01; F = 49.0324; p ≈ 4.9 × 10 ⁻ ²²⁷) and p = 7 (GCI = 5.91E-01; F = 30.675; p ≈ 2.1 × 10 ⁻ ¹⁸⁹). The effect attenuates as the horizon lengthens, still statistically significant at p = 14 (GCI = 3.07E-01; F = 6.8934; p ≈ 1.6 × 10 ⁻ ⁵⁴) and p = 20 (GCI = 1.67E-01; F = 2.4031; p ≈ 1.5 × 10 ⁻ ¹⁴), becomes marginal at p = 25 (GCI = 1.11E-01; F = 1.21038; p ≈ 0.047. In Table 3 for the Parkinson estimator, the pattern is even more pronounced when volatility is proxied with the high–low range. At p = 1 the GCI is 3.75E-01 with F = 82.2972 (p < 10 ⁻ ¹³⁹), and at p = 2 it increases to 6.19E-01 with F = 96.2247 (p < 10 ⁻ ²²⁷). The signal remains exceptionally strong at p = 5 (GCI = 8.90E-01; F = 74.7939; p ≈ 0) and p = 7 (GCI = 8.68E-01; F = 52.6472; p ≈ 0). As with vGK, significance persists at longer lags but declines smoothly: p = 14 (GCI = 6.15E-01; F = 16.3247; p ≈ 1.9 × 10 ⁻ ¹⁶⁷), p = 20 (GCI = 4.32E-01; F = 7.13324; p ≈ 5.4 × 10 ⁻ ⁸⁷), p = 25 (GCI = 3.30E-01; F = 4.03581; p ≈ 1.0 × 10 ⁻ ⁴⁵), and p = 30 (GCI = 2.64E-01; F = 2.54012; p ≈ 6.0 × 10 ⁻ ²²). Regarding Rogers–Satchell, vRS in Table 4, results align closely with the preceding estimators. For p = 1 the GCI is 3.54E-01 with F = 76.8115 (p ≈ 0); Statistical significance is observed at p = 14 (GCI = 3.56E-01; F = 8.21737; p ≈ 2.3 × 10 ⁻ ⁸⁰) and p = 20 (GCI = 1.97E-01; F = 2.8757; p ≈ 3.1 × 10 ⁻ ²¹), weakens at p = 25 (GCI = 1.27E-01; F = 1.39987; p ≈ 1.4 × 10 ⁻ ³). Across all three estimators, the largest causal signal concentrates at short lags and decays with horizon length. This pattern is economically coherent with the fast adjustment of mining activity to profitability, network conditions, and difficulty, which translates into short-run changes in electricity use that co-move with volatility. Over longer windows (beyond ~2–4 weeks), macro conditions, policy news, and shifts in investor risk appetite increasingly dominate price formation, while mining profitability tends to normalize. Hardware upgrades and efficiency improvements can also dampen the transmission from energy use to returns variability over time. Overall, the evidence supports a short-horizon, asymmetric transmission channel whereby energy-intensive mining responses help amplify near-term volatility, with diminishing influence at longer horizons.

**Table 2:** Nonlinear Granger causality test for Volatility of Bitcoin based on the Garman-Klass volatility estimator and Bitcoin-based energy consumption.

Lag parameter	CGI	F-value
	Granger causality index	
p=1	3.38E-01	72.6707***
		4.26E-125
p=2	5.35E-01	79.5368***
		2.03E-195
p=5	6.63E-01	49.0324***
		4.93E-227
p=7	5.91E-01	30.675***
		2.11E-189
p=14	3.07E-01	6.89344***
		1.58E-54
p=20	1.67E-01	2.4031***
		1.53E-14
p=25	1.11E-01	1.21038**
		0.047047
p=30	8.38E-02	0.73474
		0.9960

Notes: This table reports the nonlinear Granger causality test between Garman-Klass Volatility of Bitcoin and Bitcoin-based Electricity Consumption for a different number of lags p. The maximum number of lags considered is 30 (one month). The F-values and the p-values of the nonlinear Granger causality test are reported. The p-values of the test are given below in brackets. The null hypothesis is that the Bitcoin energy Consumption does not cause on Bitcoin Volatility. ⁎⁎⁎, ⁎⁎ and ⁎ the rejection of the null hypothesis of no-causality at the 1%, 5%, and 10% level, respectively. The Granger causality index (GCI) can be computed as $\text{log}\left(\frac{\sigma_{\text{1}}^{\text{2}}}{\sigma_{\text{2}}^{\text{2}}}\right)$.

**Table 3:** Nonlinear Granger causality test for Volatility of Bitcoin based on the Parkinson volatility estimator and Bitcoin-based energy consumption.

Lag parameter	CGI	F-value
	Granger causality index	
p=1	3.75E-01	82.2972***
		1.48E-139
p=2	6.19E-01	96.2247***
		1.34E-227
p=5	8.90E-01	74.7939***
		0.00E + 00
p=7	8.68E-01	52.6472***
		0.00E + 00
p=14	6.15E-01	16.3247***
		1.93E-167
p=20	4.32E-01	7.13324***
		5.37E-87
p=25	3.30E-01	4.03581***
		1.01E-45
p=30	2.64E-01	2.54012***
		5.97E-22

Notes: This table reports the nonlinear Granger causality test between the volatility of Bitcoin based on Parkinson estimator and Bitcoin-based Electricity Consumption for a different number of lags p. The maximum number of lags considered is 30 (one month). The F-values and the p-values of the nonlinear Granger causality test are reported. The p-values of the test are given below in brackets. The null hypothesis is that the Bitcoin energy Consumption does not cause on Bitcoin Volatility. ⁎⁎⁎, ⁎⁎ and ⁎ the rejection of the null hypothesis of no-causality at the 1%, 5%, and 10% level, respectively. The Granger causality index (GCI) can be computed as $\text{log}\left(\frac{\sigma_{\text{1}}^{\text{2}}}{\sigma_{\text{2}}^{\text{2}}}\right)$.

**Table 4:** Nonlinear Granger causality test for Volatility of Bitcoin based on the Rogers-Satchell volatility estimator and Bitcoin-based energy consumption.

Lag parameter	CGI	F-value
	Granger causality index	
p=1	3.54E-01	76.8115***
		0.00E + 00
p=2	5.67E-01	76.8115***
		0.00E + 00
p=5	7.25E-01	55.5742***
		1.01E-128
p=7	6.56E-01	35.3983***
		0.00E + 00
p=14	3.56E-01	8.21737***
		2.33E-80
p=20	1.97E-01	2.87577***
		3.14E-21
p=25	1.27E-01	1.39987***
		1.42E-03
p=30	8.86E-02	0.77973
		0.9844

Notes: This table reports the nonlinear Granger causality test between Volatility of Bitcoin based on the Rogers-Satchell estimator and Bitcoin-based Electricity Consumption for a different number of lags p. The maximum number of lags considered is 30 (one month). The F-values and the p-values of the nonlinear Granger causality test are reported. The p-values of the test are given below in brackets. The null hypothesis is that the Bitcoin energy Consumption does not cause on Bitcoin Volatility. ⁎⁎⁎, ⁎⁎ and ⁎ the rejection of the null hypothesis of no-causality at the 1%, 5%, and 10% level, respectively. The Granger causality index (GCI) can be computed as $\text{log}\left(\frac{\sigma_{\text{1}}^{\text{2}}}{\sigma_{\text{2}}^{\text{2}}}\right)$.

Bitcoin volatility is anticipated to be more significantly affected by fluctuations in Bitcoin mining activity, which directly affect electricity consumption in the short term. This is due to the rapid response of mining activity to price fluctuations, network conditions, and alterations in mining difficulty. The profitability of mining increases when Bitcoin prices rise significantly, resulting in a significant increase in electricity consumption as miners increase their operations to take advantage of the favorable market conditions. This increased mining activity can result in price fluctuations, which can contribute to an increase in volatility. Nevertheless, this dynamic effect may be restricted to brief time frames (e.g., 1–2 weeks) as miners and market participants modify their conduct. Conversely, the market may adjust to fluctuations in mining activity over extended periods (e.g., beyond 14 days), which could undermine the direct causative relationship between electricity consumption and volatility. Over time, macroeconomic trends, regulatory announcements, and fluctuations in investor sentiment become more significant determinants of Bitcoin price fluctuations. For example, the volatility of Bitcoin is more significantly influenced by broader market forces, such as global economic conditions, monetary policy, and financial market risk appetites, in the medium to long term, although electricity consumption may have a significant short-term impact. Additionally, the marginal effects of Bitcoin mining’s energy consumption on volatility may diminish as mining costs and profits stabilize over time. Bitcoin’s price volatility may decrease as mining profitability normalizes and the competitive dynamics of the mining industry modify after periods of intense energy consumption precipitated by increased mining activity. This stabilization process may account for the reason why the causal relationship between electricity consumption and volatility becomes weakened at lengthier delays. This is due to the fact that energy utilization patterns resolve into a more predictable rhythm, which no longer has a significant impact on market behavior. Furthermore, this relationship may be further weakened by improvements in mining hardware and efficacy. The direct correlation between electricity consumption and market volatility may diminish as Bitcoin mining technology advances, such as through the implementation of more energy-efficient mining devices or renewable energy sources. For example, the causal relationship between energy use and price fluctuations over time could be mitigated by improvements in energy efficiency, which could reduce the sensitivity of Bitcoin mining costs to electricity pricing.

Finally, the results in Table 4 are comparable to those of the two preceding cases. In particular, the results indicate a potential causal relationship between Bitcoin volatility, as measured by the Rogers-Satchell estimator, and Bitcoin-based Electricity Consumption Index Consumption for the majority of the delays that were observed. This further entails that the predictability of Bitcoin volatility, as measured by three volatility estimators that effectively encompass all price movements of high, opening, and closing prices, is collectively enhanced by the explanatory power of past values of Bitcoin-based energy consumption.

The economic significance of these findings extends beyond the statistical relationships identified. The short-term causal link between Bitcoin’s energy consumption and price volatility suggests that fluctuations in mining activity, driven by shifts in electricity usage, can serve as a predictive signal for market volatility. For investors, this relationship highlights the potential of using energy consumption trends as an early indicator for short-term trading strategies, enabling more informed decision-making during periods of increased mining activity or energy price fluctuations. Miners can also utilize this information to optimize their operations, balancing profitability with operational costs, particularly in response to electricity price changes. From a regulatory perspective, the findings have important implications for environmental and financial market policies. The clear short-term impact of energy consumption on volatility underscores the need for regulatory frameworks that address both the environmental footprint of Bitcoin mining and the potential risks it poses to market stability. Policies promoting the adoption of renewable energy sources or incentivizing energy-efficient mining practices could not only mitigate environmental concerns but also contribute to reducing market volatility over time. Additionally, understanding the diminishing long-term causal impact of energy consumption on volatility informs macroeconomic policy considerations, suggesting that broader economic factors eventually overshadow the influence of mining-related energy usage, necessitating a more holistic approach to cryptocurrency regulation.

Overall, the causal relationship between Bitcoin’s energy consumption and price volatility provides valuable insights into the interplay between environmental sustainability and financial stability, offering actionable information for a diverse set of stakeholders in the cryptocurrency ecosystem.

## 5. Discussion and conclusions

This study employed a non-linear Granger causality test and three volatility estimators (Gar-man-Klass, Parkinson, and Rogers-Satchell) to examine the potential causal relationship between Bitcoin volatility and Bitcoin-based Electricity Consumption. The findings suggest a causal relationship between Bitcoin volatility and electricity consumption.

Our findings of a non-linear causal relationship between Bitcoin’s energy consumption and price volatility resonate with studies on commodity markets, such as the work by Kiohos and Sariannidis [45] on gold. Both Bitcoin and gold exhibit asymmetric volatility patterns, suggesting that their market behaviors may be influenced by similar external and internal factors, including speculative trading and resource-related costs. This comparison, further supports its classification as a digital commodity with unique volatility drivers. Furthermore, statistically significant F-values were observed for a variety of delays, notably in the shorter-term range, suggesting that past Electricity Consumption levels can impact future Bitcoin volatility. These results are consistent with the relevant literature, which investigates the relationship between Bitcoin mining activity and its energy footprint (e.g., [16,18,29]).

Nevertheless, this research is the first to underscore the non-linear character of this relationship. The implementation of a non-linear Granger causality test based on artificial neural networks (ANNs) enables us to detect more intricate dynamics than traditional linear models, thereby providing a more nuanced comprehension of the impact of energy consumption on volatility. This study shows that the causal influence is of variable degrees over various time delays, with the effect diminishing over extended periods. This underscores the time-sensitive nature of energy’s impact on market behavior, a phenomenon that has not been thoroughly investigated in prior research. Enhanced mining activity, which is frequently indicated by an increase in electricity consumption, can result in a greater amount of computational power being allocated to the Bitcoin network. This could potentially affect volatility by influencing factors such as perceived scarcity and mining difficulty adjustments. The significant short-term causal relationship between electricity consumption and Bitcoin volatility has implications for market timing and trading strategies for investors. Investors who closely monitor energy consumption trends, particularly in the context of Bitcoin mining, may acquire valuable insights into potential volatility increases in the near term. This could create opportunities for short-term speculative transactions, particularly during periods of increased mining activity. The weakening of the causal link between electricity consumption and Bitcoin volatility over extended periods suggests that, while energy consumption is a critical factor in short-term price movements, other variables may be more significant in the medium to long term from a policy perspective. To improve market stability, policymakers may need to concentrate on more comprehensive regulatory and financial frameworks that address macroeconomic and speculative factors, rather than solely focusing on energy consumption. The diminishing causal relationship over time also implies that efforts to mitigate Bitcoin’s environmental impact, such as promoting the use of renewable energy or promoting more sustainable mining practices, may not directly reduce volatility in the long term. Nevertheless, the immediate term could still be stabilized by the enhancement of energy efficiency in mining, given the significant short-term link. In order to guarantee market stability and environmental protection, policymakers may need to consider a more comprehensive approach that integrates environmental sustainability initiatives with financial market supervision.

However, limitations of this study include mainly the reliance on historical data, which, while valuable for identifying past trends and patterns, may not fully capture future market dynamics or account for technological advancements that could alter the efficiency of Bitcoin mining operations. The rapid evolution of mining hardware and shifts in regulatory or economic environments may significantly influence the relationship between energy consumption and price volatility in ways that historical data cannot anticipate. Furthermore, the accuracy of the energy consumption data, derived from the Cambridge Bitcoin Electricity Consumption Index, may be influenced by estimation biases. These biases could arise from heterogeneous factors such as differences in mining hardware efficiency, the geographic dispersion of mining activities, and the varying reliance on renewable versus non-renewable energy sources across regions. Such factors introduce potential variability into the energy consumption estimates, which may affect the robustness and generalizability of the study’s findings.

Such findings imply that the bivariate VARNN framework may be susceptible to omitted variable bias and that the model’s flexibility could be extended further with additional informative inputs. Ultimately, this highlights the importance of careful variable selection and offers an avenue for future research. Extending the VARNN to a multivariate causality framework or hybridizing it with variance-based causality tests—such as the approach proposed by Chang and McAleer (2017) [4]—could reveal deeper structural dependencies among electricity consumption, speculation, and volatility in cryptocurrency markets. While our current framework captures predictive non-linear dependencies, future work could explore causality in volatility more formally by adopting models based on conditional variance structures.

Overall, our results have direct value for Bitcoin investors as risk-management inputs rather than return-forecasting signals: short-horizon links between electricity consumption and volatility can be used to monitor risk, adjust leverage/position size, and set tighter drawdown or VaR limits during energy-driven stress. Practically, a rising energy-volatility signal flags periods to de-risk or hedge (e.g., via options), whereas muted signals justify baseline exposure.

Future research should incorporate additional determinants of Bitcoin volatility, such as regulatory changes, investor sentiment, and global macro-financial conditions, to assess their incremental and interacting effects. Our study contributes by highlighting a potential causal link between Bitcoin-based electricity consumption and volatility, advancing the evidence on energy–market dynamics in crypto. While the findings are suggestive, further work is needed to analyse the underlying mechanisms, test regime-specific behavior, and strengthen identification and robustness.
